# Variability in Vulnerability Assessment of Older People by Individual General Practitioners: A Cross-Sectional Study

**DOI:** 10.1371/journal.pone.0108666

**Published:** 2014-11-07

**Authors:** Yvonne M. Drewes, Jeanet W. Blom, Willem J. J. Assendelft, Theo Stijnen, Wendy P. J. den Elzen, Jacobijn Gussekloo

**Affiliations:** 1 Leiden University Medical Center, Department of Public Health and Primary Care, Leiden, The Netherlands; 2 Radboud University Nijmegen Medical Centre, Department of Primary Community Care, Nijmegen, the Netherlands; 3 Leiden University Medical Center, Department of Medical Statistics and Bioinformatics, Leiden, The Netherlands; Medical University Vienna, Austria

## Abstract

**Background:**

In clinical practice, GPs appeared to have an internalized concept of “vulnerability.” This study investigates the variability between general practitioners (GPs) in their vulnerability-assessment of older persons.

**Methods:**

Seventy-seven GPs categorized their 75-plus patients (n = 11392) into non-vulnerable, possibly vulnerable, and vulnerable patients. GPs personal and practice characteristics were collected. From a sample of 2828 patients the following domains were recorded: sociodemographic, functional [instrumental activities in daily living (IADL), basic activities in daily living (BADL)], somatic (number of diseases, polypharmacy), psychological (Mini-Mental State Examination, 15-item Geriatric Depression Scale; GDS-15) and social (De Jong-Gierveld Loneliness Scale; DJG). Variability in GPs' assessment of vulnerability was tested with mixed effects logistic regression. P-values for variability (p_var_) were calculated by the log-likelihood ratio test.

**Results:**

Participating GPs assessed the vulnerability of 10,361 patients. The median percentage of vulnerable patients was 32.0% (IQR 19.5 to 40.1%). From the somatic and psychological domains, GPs uniformly took into account the patient characteristics ‘total number of diseases’ (OR 1.7, 90% range  = 0, p_var_ = 1), ‘polypharmacy’ (OR 2.3, 90% range  = 0, p_var_ = 1) and ‘GDS-15’ (OR 1.6, 90% range  = 0, p_var_ = 1). GPs vary in the way they assessed their patients' vulnerability in the functional domain (IADL: median OR 2.8, 90% range 1.6, p_var_<0.001, BADL: median OR 2.4, 90% range 2.9, p_var_<0.001) and the social domain (DJG: median OR 1.2, 90% range  = 1.2, p_var_<0.001).

**Conclusions:**

GPs seem to share a medical concept of vulnerability, since they take somatic and psychological characteristics uniformly into account in the vulnerability-assessment of older persons. In the functional and social domains, however, variability was found. Vulnerability assessment by GPs might be a promising instrument to select older people for geriatric care if more uniformity could be achieved.

**Trial Registration:**

Netherlands Trial Register NTR1946

## Introduction

In aging societies the prevalence of vulnerability increases [1]. The vulnerable older population is described as the group of older people that presents the most complex and challenging problems to physicians and other healthcare professionals and often require geriatric care [2]. Frailty and vulnerability are terms widely used in discussions on older people, in policy documents and in daily care. The frailty phenotype was introduced by Fried et al. and was defined as meeting three or more of the following criteria: unintentional weight loss, self-reported exhaustion, slow walking speed, weak grip strength, and low physical activity level [3]. The term vulnerability indicates a more heterogeneous group of older people with multiple chronic conditions and/or loss of function in one or more domains (e.g. functional, somatic, psychological and social domains) [4], [5]. Despite the development of various tools to screen for manifestations of vulnerability [6]–[11], no standardized and valid method to assess vulnerability is currently available. Nevertheless, physicians, especially general practitioners (GPs), appear to be able to work with an implicit concept of vulnerability [12], [13]. Probably most general practitioners are aware of the existence of this subset of older patients who are vulnerable intuitively, without measuring the specific characteristics of frailty.

However, it is unknown whether these implicit concepts of vulnerability are uniform. GPs may share a unique perspective on what defines vulnerability, but may also have distinct perspectives on vulnerability. If and when the implicit vulnerability concepts of GPs appear to be identical, assessment by GPs can be a promising instrument to select older people for specific geriatric care, because such assessment is relatively simple, fast and inexpensive. Therefore, the present study investigates the variability between GPs in their vulnerability assessment of older people, to determine whether GPs share a uniform concept of vulnerability.

## Materials and Methods

### Study design and recruitment

The present analysis is embedded in the Integrated Systematic Care for Older People (ISCOPE) study, a cluster-randomized controlled trial to investigate the effect of pro-active care for patients aged 75 years and over.

GPs working in the northern part of the South Holland province were recruited to participate in this study. During the inclusion period (September 2009-September 2010), all patients aged ≥75 years in the participating practices received an invitation (by mail) from their GP to participate in the study. Excluded from the study were persons with a terminal illness or a life expectancy of ≤3 months.

Participants were asked to complete a postal screening questionnaire for complex health problems on four domains of health (functional, somatic, psychological and social); the questionnaire was sent together with the invitation. For logistic reasons, participants were randomly selected for an interview at home to obtain baseline data on sociodemographic, functional, somatic, psychological and social characteristics; 15% of the participants with problems on 0 or1 domains of the postal screening questionnaire were visited, 60% of the participants with problems on 2 domains were visited and all participants with problems on 3 or 4 domains were visited. The visits were carried out by research assistants.

Written informed consent was obtained from all participants; for participants with severe cognitive impairment, informed consent was obtained from a proxy.

The Medical Ethical Committee of the Leiden University Medical Center approved the study in 2009.

### Primary outcome

Before sending the questionnaires to the patients, GPs were asked to assess the vulnerability of all their patients aged ≥75 years in three categories: i) not vulnerable, ii) possibly vulnerable, and iii) vulnerable. Since our goal was to assess patient vulnerability as defined by the GPs themselves, GPs were not provided with a specific definition of vulnerability. Instead, they were asked to indicate ‘in their opinion’ which of their patients were considered vulnerable in the context of this study.

### Determinants

#### GP characteristics

From the GPs we collected information on personal characteristics (sex and age), practice characteristics [practice type (single-handed or group), urbanization level (rural or urban)] and characteristics of their older patients (number of patients aged ≥75 years, median age of these patients, and percentage of males).

#### Patient characteristics

From interviews during home visits we obtained information on sociodemographic characteristics (age, sex, income, living situation and home situation). In addition, the presence of problems in four domains of health was assessed with questionnaires.

In the *functional* domain, disability was assessed with the Groningen Activity Restriction Scale (GARS) questionnaire [14], [15] which assesses disabilities in competence in nine instrumental activities in daily living (IADL) and in nine basic activities in daily living (BADL). Scores on the IADL and BADL range from 9–36 points with higher scores indicating poorer performance.

In the *somatic* domain information was assessed on self-reported polypharmacy (i.e. taking at least four drugs) and on the medical history covering 17 diseases: diabetes mellitus, heart failure, obstructive lung disease, incontinence, arthritis, osteoporosis, dizziness and falls, prostate problems, cognitive decline, hearing disorder, visual disorder and (a history of) stroke, malignancy, fracture, myocardial infarction, depression and anxiety.

In the *psychological* domain cognitive function was evaluated with the Mini-Mental State Examination (MMSE) [16]; scores range from 0–30 points with lower scores indicating poorer cognitive performance. Depressive symptoms were assessed with the 15-item Geriatric Depression Scale (GDS-15) [17], which is specifically developed to screen for depressive symptoms in older people; scores range from 0 (optimal) to 15 points.

In the *social* domain, the De Jong-Gierveld Loneliness Scale (DJG) was used to assess feelings of loneliness, with higher scores (range 0–11) indicating more severe loneliness [18], [19]. The GDS-15 and the DJG were restricted to those with a MMSE score of 19 or higher.

### Statistical analysis

To describe the GPs' personal/practice characteristics and patient populations, the median and interquartile range (IQR) was calculated of their total population aged ≥75 years.

For the analysis of vulnerability, the assessments by GPs were dichotomized into ‘not/possibly vulnerable’ and ‘vulnerable’. The outcome ‘unknown’ (2.6% of the total population) was handled as missing data.

In the populations assessed as ‘not/possibly vulnerable’ or ‘vulnerable’ the association between GP characteristics and percentage of vulnerable older persons per GP was examined. The median percentage (IQR) of vulnerable older persons per GP characteristic was calculated and differences analyzed with the Mann-Whitney U-test.

To describe the characteristics of the sample that was visited at home, characteristics of the ‘not/possibly vulnerable’ and ‘vulnerable’ persons were compared by testing differences in medians (IQR) for continuous variables with the Mann-Whitney U-test, and differences in proportions for dichotomous variables with Pearson's chi-square test.

To investigate the association between patient characteristics and the patient's chance to be assessed as vulnerable, we applied mixed effects logistic regression on the participants visited at home and who were assessed as ‘not/possibly vulnerable’ or ‘vulnerable’. To adjust for the intra-class correlation within the practices a random intercept was used. The strength of an association was expressed by the odds ratio (OR) for dichotomous variables and by the OR per standard deviation (SD) for continuous variables. If one GP gives more weight to a characteristic than another GP, the ORs will vary between GPs. To investigate whether the OR for a certain characteristic varies between GPs, a subsequent analysis was performed in which we extended the random intercept model with an extra random term for that characteristic, thereby allowing that every GP has his/her own OR. The model assumes a lognormal distribution for the GP-specific ORs. The median and 90% reference interval of this distribution were estimated. The reference interval runs from the 5^th^ to the 95^th^ percentile of the distribution and contains the ORs of 90% of the GPs, thereby serving as a characterization of the variability between GPs in the weight they attribute to a patient characteristic in assessing vulnerability. If the GPs do not vary in their assessment, their ORs will be the same and there will be no range around the median OR. If the GPs vary in their assessment and the range includes OR = 1, some GPs will weigh a patient characteristic in a direction opposite to that of other GPs. P-values for variability (p_var_) are calculated with the log-likelihood ratio test.

All analyses were performed with IBM SPSS Statistics version 20.

## Results

### Study population and vulnerability assessment

In total, 77 GPs in 55 participating general practices worked for 11,392 registered patients aged 75 years and over.

Table 1 presents the baseline characteristics of these 77 GPs. Their median age was 51.4 (IQR 43.1 to 57.1) years and 46 (59.7%) were male. The majority of the GPs (77.9%) worked in an urban environment. The number of registered patients aged ≥75 years per GP ranged from 12 to 479 with a median of 131 (IQR 66 to 210).

**Table 1:** Personal and practice characteristics of GPs (n = 77) and the characteristics of their registered population aged 75 years and over.

	n (%) or median (IQR)
*Characteristics of general practitioner (n = 77)*	
Age in years	51.4 (43.1–57.1)
Sex (male)	46 (59.7)
Environment (urban)	60 (77.9)
Type of practice (single-handed)	24 (31.2)
*Characteristics of the population per GP*	
Number of persons aged 75 years and over	131 (66–210)
Median age of the population	80.8 (79.8–82.1) *
Percentage male in the population aged 75 years and over	36.4 (31.9–40.0)†
Percentage vulnerable older persons‡	32.0 (19.5–40.1)†

*median (IQR) of the median ages per GP-population.

† median (IQR) of the percentages per GP-population.

‡ calculated for 10,361 people who were assessed by the GP as not vulnerable/possibly vulnerable/vulnerable.

**Figure 1:**
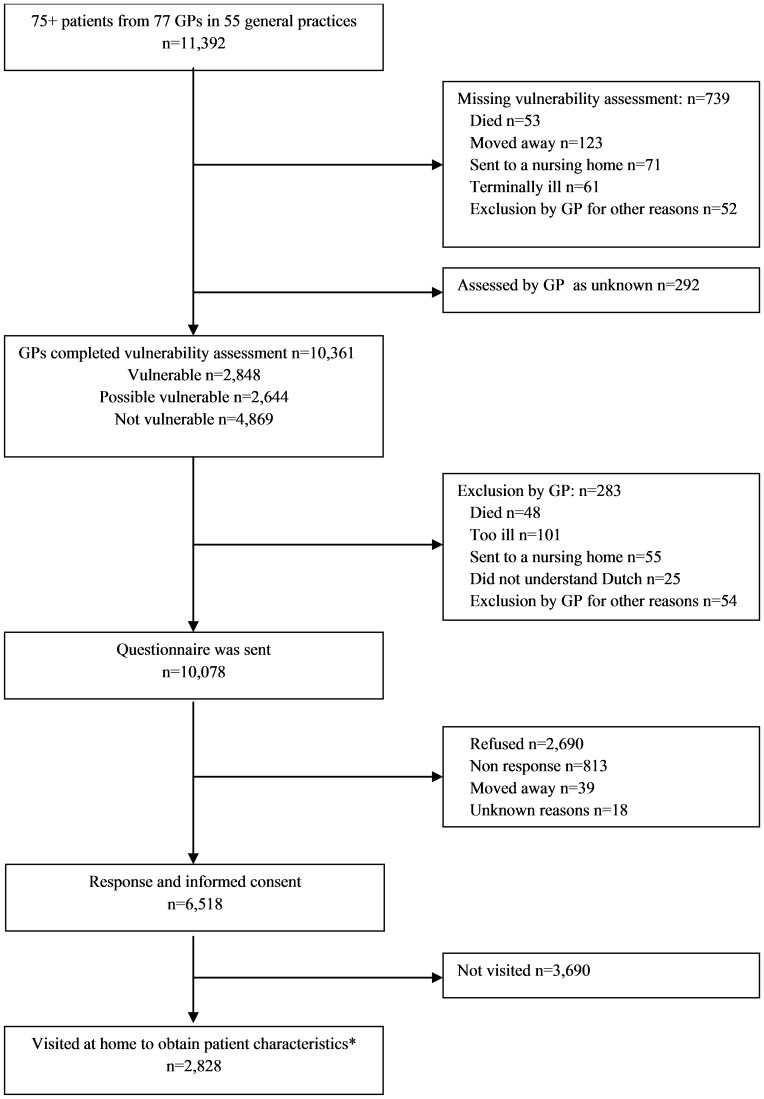
Flowchart from the start of the study with all registered patients of 77 general practitioners till the selection visited at home. *Selection based on the outcomes of the questionnaire: 15% of the participants with problems on 0 or1 domains of the questionnaire were visited, 60% of the participants with problems on 2 domains and all participants with problems on 3 or 4 domains.

Of the 11,392 eligible patients, the GPs completed the assessment for 10,361 patients (see flowchart in Figure 1). Of the latter group, 2,848 (27.5%) were rated as vulnerable, 2,644 (25.5%) as possibly vulnerable, and 4,869 (47.0%) as not vulnerable. Of the remaining 1031 persons, 292 were assessed as ‘unknown’ and 739 assessments were missing (reasons for missing: 53 persons died, 123 persons moved away, 71 persons were sent to a nursing home, 61 persons were terminally ill, 52 persons were excluded for other reasons, and 379 were missing for unknown reason). Overall, the median percentage of vulnerable patients per GP was 32.0% (IQR 19.5 to 40.1%), ranging from 2.4% to 81.0%.

GPs living in an urban environment assessed a higher percentage of their patients as vulnerable than GPs in a rural environment (33.3% (IQR 22.1 to 40.9%) vs. 23.6% (IQR 8.4 to 37.4%), p = 0.044). No differences in vulnerability assessment were found for the type of practice, for the GPs' personal characteristics (age and sex) and for the number of persons aged ≥75 years in their practice (all p>0.05) (Table 2).

**Table 2:** Association between characteristics of 77 GPs and the percentage of vulnerable older persons per practice (n = 10,361).

Characteristics of GPs and their practice		n	Median percentage of vulnerable older persons	p-value*
GPs' age	≤50 years	35	35.7 (23.5–43.2)	0.091
	>50 years	42	28.8 (15.5–38.4)	
GPs' sex	Male	46	32.0 (19.7–38.8)	0.705
	Female	31	33.3 (19.1–40.8)	
Environment	Urban	60	33.3 (22.1–40.9)	0.044
	Rural	17	23.6 (8.4–37.4)	
Type of practice	Single-handed	24	26.2 (10.5–35.7)	0.052
	Group	53	33.6 (22.8–40.8)	
Total number of 75-plus persons in practice	≤130 persons	38	34.2 (22.2–42.9)	0.121
	>130 persons	39	30.2 (16.1–36.4)	

*Mann-Whitney U-test.

Of the 10,361 patients assessed by the GPs, a questionnaire was sent to 10,078 patients (excluding: 48 persons who died, 101 persons too ill, 55 persons in a nursing home, 25 persons who did not understand Dutch, and 54 persons were excluded by the GP for other reasons). Of these, 6518 (64.7%) persons responded to the questionnaire (reasons for non-participation: 2690 refused, 813 did not respond, 39 moved away, and 18 for other unknown reasons).

Based on the outcomes of the questionnaire, a sample of 2828 persons was visited at home to obtain patient characteristics. Table 3 presents the characteristics of these persons of whom 31.6% (n = 894) were assessed as vulnerable and 68.4% (n = 1934) as not/possibly vulnerable. The participants assessed as vulnerable were older, i.e. median age 83 years (IQR 80 to 88 years) vs. 81 years (IQR 78 to 86) (p<0.001) and were more often living in a residential home (15.4% vs. 7.8%) (p<0.001). In all health domains (functional, somatic, psychological and social) the vulnerable older people had less favorable scores than the not/possibly vulnerable persons (all p<0.001).

**Table 3:** Association between patient characteristics and vulnerability assessment by the GP (n = 2828).

	Vulnerability by GP		p-value*
	Yes	Not/possibly	
	(n = 894)	(n = 1934)	
	n (%) or median (IQR)	n (%) or median (IQR)	
*Socio-demographic factors*			
Age	83 (80–88)	81 (78–86)	<0.001
Sex, male	294 (32.9)	598 (30.9)	0.296
Income, low (only state pension)†	146 (16.4)	273 (14.1)	0.116
Living situation, living alone†	580 (64.9)	1204 (62.3)	0.184
Home, residential†	138 (15.4)	150 (7.8)	<0.001
*Functional domain*			
IADL†	27 (20–33)	19 (13–25)	<0.001
BADL†	11 (9–17)	9 (9–11)	<0.001
*Somatic domain*			
Total number of self reported diseases	5 (3–6)	4 (2–5)	<0.001
Self reported poly-pharmacy (≥4 drugs)†	673 (75.7)	1143 (59.2)	<0.001
*Psychological domain*			
MMSE†	27 (24–29)	28 (26–29)	<0.001
GDS-15† ‡	2 (1–4)	1 (0–3)	<0.001
*Social domain*			
DJG† ‡	3 (1–5)	2 (0–4)	<0.001

*Mann-Whitney U-test for continuous variables or Pearson's chi-square test for dichotomous variables.

IADL  =  Instrumental Activities in Daily Living, BADL  =  Basic Activities in Daily Living, MMSE  =  Mini-Mental State Examination, GDS-15 = 15-item Geriatric Depression Scale, DJG  =  De Jong-Gierveld Loneliness Scale.

† missing in 1–38 participants.

‡ not administered in 149 participants.

### Variability between GPs in vulnerability assessment

**Figure 2:**
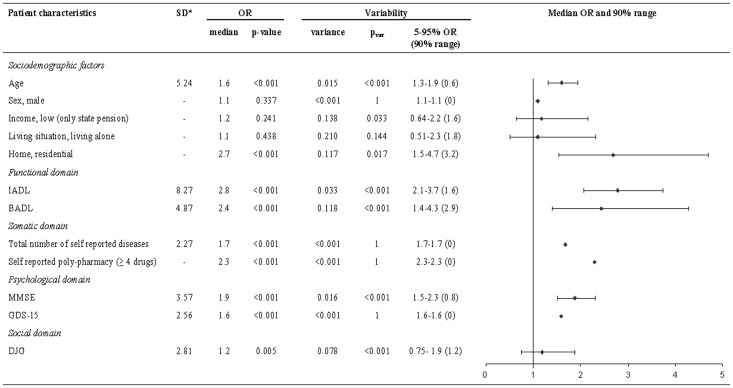
Variability in the influence of patient characteristics on the vulnerability assessment by the GP (n = 2828). *Standard deviation (SD) is calculated for continuous data. The OR is estimated per SD decline in functioning. IADL  =  Instrumental Activities in Daily Living, BADL  =  Basic Activities in Daily Living, MMSE  =  Mini-Mental State Examination, GDS-15 = 15-item Geriatric Depression Scale, DJG  =  De Jong-Gierveld Loneliness Scale.

To investigate the variability between GPs in the weight they attribute to a patient characteristic in assessing vulnerability, data of the 2828 participants visited at home were analyzed (Figure 2).

In the sociodemographic domain, GPs only used age and residential living in their vulnerability assessment and differed in the weight they attributed to these factors (median OR 1.6, 90% range  = 0.6, p_var_<0.001 and median OR 2.7, 90% range  = 3.2, p_var_ = 0.017, respectively).

Variability was also found between GPs in the functional domain (IADL: median OR 2.8, 90% range  = 1.6, p_var_<0.001 and BADL: median OR 2.4, 90% range  = 2.9, p_var_<0.001).

No variability was found in the somatic domain (self-reported diseases median OR 1.7, 90% range  = 0, p_var_ = 1, polypharmacy median OR 2.3, 90% range  = 0, p_var_ = 1).

In the psychological domain variability was also absent for depressive symptoms (GDS-15: median OR 1.6, 90% range  = 0, p_var_ = 1), but GPs differed in the weight they attributed to cognition (MMSE: median OR 1.9, 90% range  = 0.8, p_var_<0.001).

In the social domain, variability was found between GPs and they also differed in the direction of the association with vulnerability: i.e. some GPs gave a positive and others a negative weight to loneliness (DJG: median OR 1.2, 90% range  = 1.2, p_var_<0.001).

## Discussion

### Principal findings

This study investigated the variability in vulnerability assessments by GPs. The percentage of older patients assessed as ‘vulnerable’ by the GP varied per practice (median 32.0%, IQR 19.5 to 40.1%, range 2.4 to 81.0%). This variation was not only due to differences in the patient-populations of the GPs, but also depended on differences in the weight GPs attributed to some patient characteristics in the vulnerability assessment. All GPs took some somatic and psychological characteristics uniformly into account. In the functional and social domains variability was found in the way GPs assessed their patients' vulnerability.

In the present study, an urban environment was the only practice characteristic that was associated with the percentage of vulnerable older persons. However, the environment of the GP's practice can also be considered as a patient characteristic. Apparently, older persons living in a city were more likely to be assessed as ‘vulnerable’.

In the somatic and psychological domains, patient characteristics predicted the vulnerability assessment by the GP, and the GPs weighed these patient characteristics almost equally. The formal education of GPs and their corresponding focus on diseases may explain these findings. GPs are educated in clinical observation, which mainly takes into account somatic and psychological characteristics, as well as age and sex. This study shows that (with the exception of sex) GPs almost uniformly attribute predictive values to these items; sex did not predict the outcome of the vulnerability assessment and no differences were found between GPs. Apparently, sex is not a discriminative factor in the vulnerability assessment by GPs.

In the functional domain, although strong predictive values of the patient characteristics were found, GPs differed in the way they used these patient characteristics in their assessment. Although GPs are aware of the importance of functional status for vulnerability, they might not use a standard approach while taking functioning into account. Similar results were found for type of residence: this might be explained by the fact that people in residential homes often have a functional impairment. In clinical practice, GPs tend to focus on medical problems using a disease model as concept of care. If the vulnerability assessment was carried out, for example, by nurses (who are mainly trained in functional models), it is likely that uniform outcomes will be found in the functional domain and variability in the somatic domain. If GPs received more training in the use of functional models [20], the differences between them might become smaller.

Finally, in the social domain the loneliness score predicted vulnerability, even though GPs varied in the way they took this characteristic into account: i.e. loneliness increased a patient's chance to be assessed as vulnerable by most GPs, whereas some GPs weighed loneliness in the opposite direction. Interpretation of this outcome is difficult but might indicate that GPs are unaware of patients' loneliness as measured in the present study; this is in line with earlier research indicating that some GPs rarely ask their patients about loneliness [21]. The impact of the social domain on vulnerability should be explored in further studies.

### Comparison with existing literature

Various tools have been developed to screen for vulnerability [5]–[11], but no standardized and valid method is available. Knowledge on the prevalence of vulnerability as assessed by GPs is currently limited. For example, although Hoogendijk et al. compared clinical judgment with several frailty instruments, only one GP was involved [22]. To our knowledge, ours is the first study in which several GPs were asked to assess the vulnerability of their registered older patients, without imposing a pre-described definition. The present study shows that GPs generally share a unique perspective on what defines vulnerability in the somatic and psychological domain, but differ in the way they interpret the functional domain. GPs are probably not aware of this phenomenon, because other studies report that they consider themselves able to work with an undefined concept of vulnerability [12], [13].

### Strengths and limitations

The present study has several strengths. It is a large population-based study of GPs and their registered patients aged ≥75 years. Also, because we did not impose a definition of ‘vulnerability’, the assessment revealed the GP's own interpretation of the concept of vulnerability. In this study, 77 GPs assessed the vulnerability of almost all of their patients; therefore, there was no (or minimal) selection of patients in the assessment by the GP. Furthermore, GPs were unaware of the outcome of the study measurements because they performed the assessments before randomization and before other data were collected.

Unfortunately, it was not possible to interview the GPs about the concepts of vulnerability they used during their assessments. However, we were able to analyze the variability between GPs in the way they weighed the characteristics of their patients. In this study the variability between GPs might be an overestimation of the true variability, because the most obviously vulnerable patients may not have been included in our analysis.

Variability can also partly be explained by the fact that GPs might not have examined all their patients recently. At the moment of assessing their patients' vulnerability, GPs were unable to take all recent changes in patients' functioning into account. Moreover, other patient characteristics that might influence the vulnerability assessment may not have been examined. However, the literature does not include any other important characteristics that we did not investigate.

### Implications and further research

GPs appear to share a medical concept of vulnerability because they take somatic and psychological characteristics uniformly into account in the vulnerability assessment; however, they differ in the weight they attribute to functional status and loneliness. More uniformity might be achieved if GPs receive training in the use of a functional model as concept of care. The impact of the social domain on vulnerability should be explored in further studies. More research is needed to investigate the appropriateness of the vulnerability assessment by GPs, by comparing the outcomes of their vulnerability assessment with those of other tools that measure vulnerability. Such analyses may reveal the additional value of screening tools compared to assessment by GPs, which is a simple, inexpensive and apparently reliable method. If stratification on vulnerability becomes feasible, this will facilitate the selection of older individuals who may best benefit from specific geriatric care.
